# Designing an AI companion to support informal caregivers in role transition: insights from a design science approach

**DOI:** 10.1186/s12912-025-03868-2

**Published:** 2025-09-10

**Authors:** David Walter, Jennifer Pengel, Paul-Ferdinand Steuck, Marco Di Maria, Ralf Knackstedt, Anne Meissner

**Affiliations:** 1 Institute of Business Administration and Business Informatics, Information Systems and Enterprise Modeling, University of Hildesheim, Hildesheim, Germany; 2 Institute of Business Administration and Business Informatics, IT for the Caring Society, University of Hildesheim, Hildesheim, Germany

**Keywords:** Informal care, Artificial intelligence, Generative AI, Chatbot, Role awareness, Design science research

## Abstract

**Background:**

As populations age, informal caregivers play an increasingly vital role in long-term care, with 80% of care provided by family members in Europe. However, many individuals do not immediately recognize themselves as caregivers, especially in the early stages. This lack of awareness can increase physical and emotional stress and delay access to support services. The phenomenon of *hidden care*, where substantial care is provided without formally acknowledging the role, further exacerbates these issues. To address this, we developed an AI-driven chatbot designed to support informal caregivers recognize their role, reflect on their situation, and identify relevant support options. This paper explores how an AI-based chatbot can be designed to support informal caregivers in reflecting on and re-evaluating their caregiving roles.

**Methods:**

Following a design science research approach, we evaluate the chatbot design via focused semistructured interviews and think-aloud sessions with informal caregivers to assess its utility, completeness and potential for supporting role transitions through the lens of unlearning. The data were analyzed via Braun and Clarke’s thematic analysis.

**Results:**

The chatbot has the potential to support caregivers in recognizing their role and reflecting on their experiences, with participants reporting increased self-awareness triggered by reflective prompts and recommendations of useful personalized support resources. Seven initial design principles for AI-based chatbot development in transitional informal care contexts were identified. These principles emphasize personalized assessment, transparent information, role awareness support, accessibility, and continuous companionship.

**Conclusions:**

This study demonstrates the potential of AI-driven chatbots to support informal caregivers during critical role transitions. Future research should build on these insights to design context-aware solutions that responsibly embed AI into caregiving realities.

**Clinical trial:**

No clinical trial.

**Supplementary Information:**

The online version contains supplementary material available at 10.1186/s12912-025-03868-2.

## Background

Aging and illness can generate significant care demands [1], which are typically met by informal caregivers, who share a personal relationship with the person in need of care and provide practical, emotional, and organizational support [2]. Care needs may develop gradually or become acute due to sudden events. In any case, they disrupt daily routines and necessitate immediate decisions [3]. Most significant others are unprepared to manage either the scenario or the associated care arrangements [4], resulting in caregiver burden, such as physical or emotional strains [5].

The transition to informal caregiving involves adopting a caregiving identity, which includes acquiring new responsibilities as well as unlearning, defined as the conscious or unconscious letting go of prior routines, assumptions, or self-perceptions that no longer match the demands of the caregiving role [6–8]. A lack of role awareness, commonly observed in *hidden caregivers*, often hinders individuals from recognizing their caregiving situation and delays the uptake of available support services [9]. Moreover, misconceptions, such as the belief that care recipients must be completely dependent to qualify for support, can further delay help-seeking [4]. These challenges are well documented, e.g., [10–12].

To conceptualize the transition into informal caregiving, we draw on the Family Resilience Framework [13]. It was developed to understand how families adapt to adversity and conceptualizes this adaption not as a linear or cyclical sequence of phases, but as a developmental, systemic process unfolding across three interrelated domains: shared belief systems, organizational patterns, and communication/problem-solving practices. Based on this framework, we understand the transition to caregiving as a recursive and evolving process, often triggered by acute role disruptions, that prompts the recognition of new support needs and extends into sustained realignment across family belief systems, organizational routines, and communication patterns.

Successful navigation of these transitions, as research in care and related fields has shown, often requires not only adaptation but also unlearning [14, 15]. Unlearning refers to the process by which individuals disengage from previous role identities and routines to realign themselves with new forms of responsibility and identity. Similar to the recursive nature of family resilience processes, unlearning is not a one-off event, but unfolds iteratively through phases of tension, adjustment, and reorientation. Key to this process are moments of “destabilization,” where familiar routines or identities are disrupted [16], for instance, by a shift in the care situation or the realization of new responsibilities, which create opportunities for reflection and transformation. Such moments of destabilization can act as catalysts for reflection [17]. In this context, reflection is understood as a deliberate meaning-making process that enables caregivers to critically examine emerging challenges, question previous assumptions, and realign actions with the new role demands [18].

Despite solid theoretical foundations, design knowledge on how to design effective support systems for informal caregiving remains scarce. While (generative) AI and chatbots have been explored in professional care [19, 20] research on informal caregiving has focused on, e.g., educational tools [21] and burden reduction [22] rather than on supporting role transitions and reflection.

To address this gap, we developed a chatbot [23] that accompanies informal caregivers during this adaptation process. Currently, the chatbot is intended for one-time use. However, given that role transitions often extend over longer periods [12], we aim to incorporate companionship features, understood not merely as repeated interactions but also as a set of design elements that enable the system to offer empathetic, context-aware, and trust-building support over time [24]. These features are intended to foster an ongoing sense of support by enabling the chatbot to remember prior interactions, resonate emotionally, and create a perceived relationship with the user. Although our chatbot’s design was informed by design knowledge, there is still a lack of empirical research validating such approaches for fostering reflection and role awareness specifically among informal caregivers. This highlights the need for further investigation into design knowledge that effectively support these processes. Accordingly, our research question is: *How should an AI companion be designed to support informal caregivers in transitioning into their caregiving role and in accessing relevant support services?*

## Methods

### Aim, design and setting

**Fig. 1:**
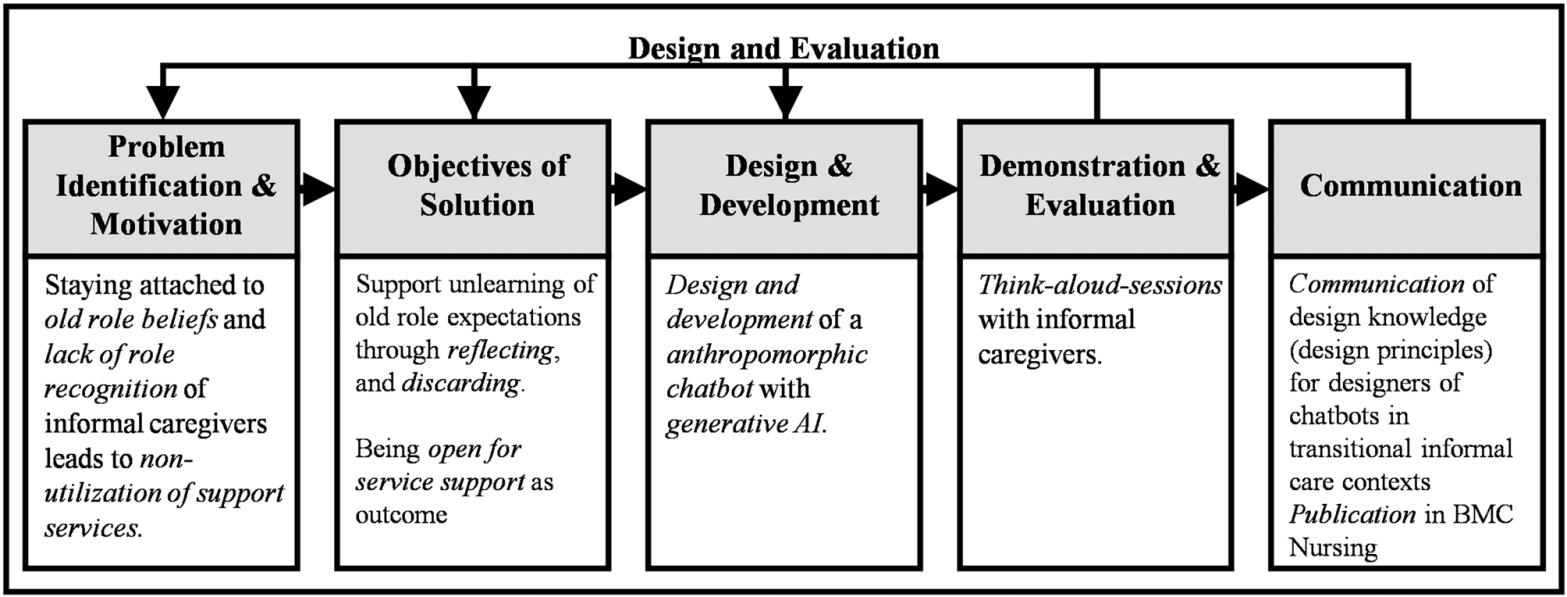
Phases of the design science research project

To evaluate our chatbot and derive relevant design knowledge in the form of design principles (DPs), an exploratory design science research (DSR) approach was utilized [25]. DPs, as a form of codified design knowledge, serve to build a cumulative knowledge base for the development of specific classes of IT artifacts [26]. The project was structured using the design science research methodology (DSRM), which encompasses the phases of problem identification and motivation, objective definition, design and development, demonstration and evaluation, and communication [27]. As a pilot study, this work aims to generate initial insights into design recommendations. An overview of the DSR project is presented in Fig. 1. The first three phases were reported in a previous study [23].

Regarding the *problem identification and motivation phase*, we address the issue of limited role recognition among informal caregivers and the persistence of outdated role beliefs, which together contribute to the underutilization of support services such as caregiving contact points [4, 28]. Based on the Family Resilience Framework [13], our approach addresses key family adaptation processes such as joint meaning-making, flexible role reorganization, and collaborative problem-solving, which become especially relevant as caregiving responsibilities emerge and intensify.

In the *objectives for a solution phase*, we derived design goals from existing propositions for informal care apps [29] and requirements for unlearning support systems [30], selecting only those directly relevant to our identified problem. A chatbot can function as a trigger, supporting informal caregivers in unlearning obsolete role beliefs and facilitating the transition to the critical choice phase, ultimately increasing the uptake of available support services. Our initial prototype therefore explicitly targets the illness appraisal, acute response, and critical choice phases of the caregiving journey. In the *design and development phase*, we instantiated the identified requirements into a chatbot.

This study focuses on the demonstration and evaluation of the chatbot prototype. For *demonstration and evaluation* [31], our prototype of the chatbot was used by seven informal caregivers in think-aloud sessions [32]. We assessed its perceived utility, understandability of features and completeness. In addition, we examined whether and how reflection processes regarding caregivers’ roles were triggered during chatbot use. For the *communication phase*, seven design principles were derived and formulated. As a pilot study, this work seeks to provide initial insights for design recommendations. Our study was reported in accordance with the Consolidated Criteria for Reporting Qualitative Research (COREQ) [33].

### Intervention

The generative AI-based chatbot prototype, which was designed in a previous study [23], aims to encourage informal caregivers to reflect on their caregiving role, let go of (i.e., unlearning) prior belief and behavior, and subsequently provide personalized connections to relevant support services. The chatbot was developed via OpenAI’s GPT-4 model with prompt engineering (i.e., the design of input instructions to elicit controlled outputs), following a predefined flow, and a retrieval-augmented generation (RAG) setup. The RAG system enables the chatbot to suggest relevant support services by retrieving information from a curated database of caregiving resources by the research team. The chatbot does not store user data beyond the session; however, user interactions via ChatGPT are stored by OpenAI for up to 30 days. The chatbot’s design features were based on design requirements derived from the literature reviews on informal care support systems [29], unlearning support systems [30], and human-like chatbot design [34].

**Fig. 2:**
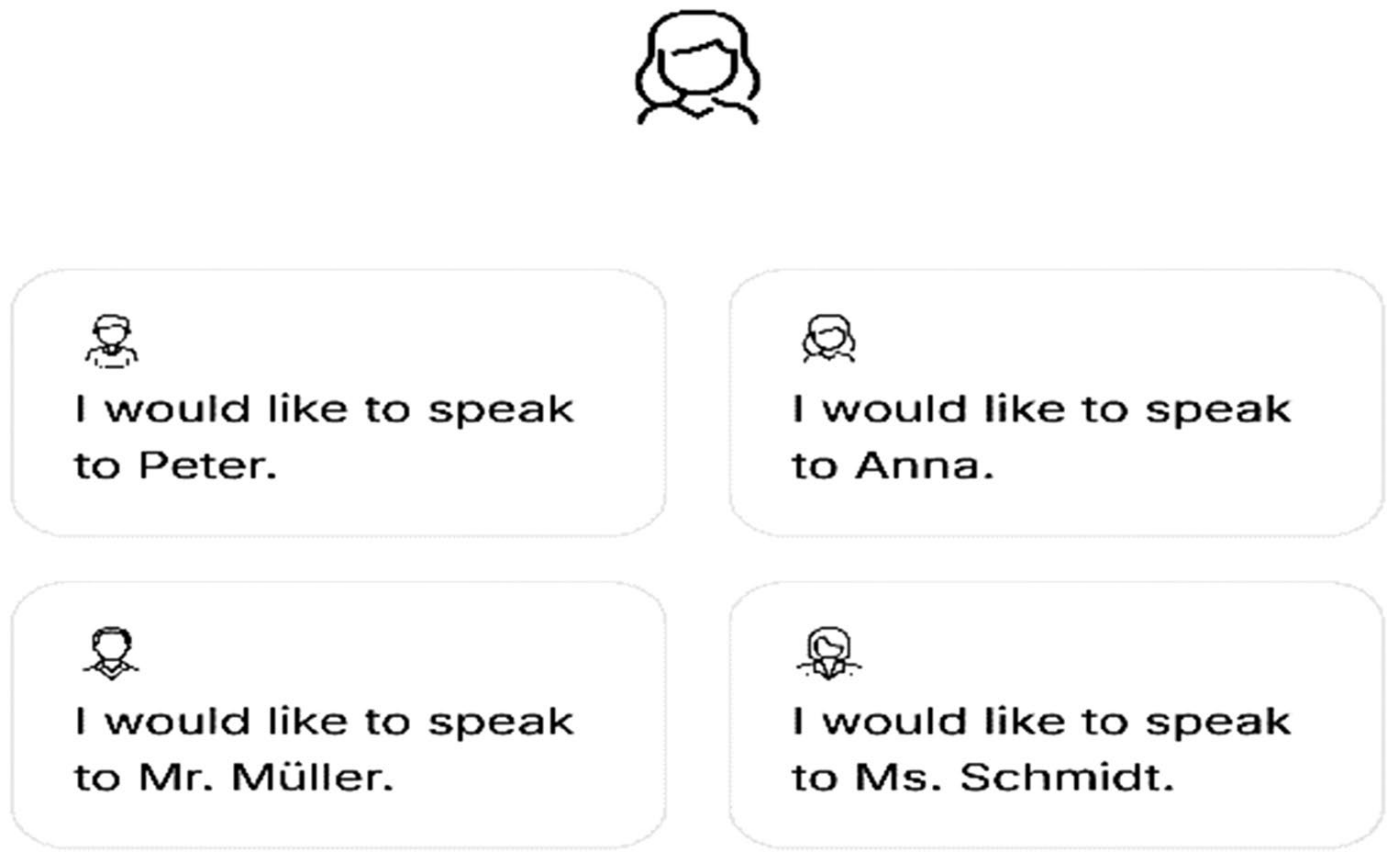
Persona selection in the chatbot

The interaction begins with users selecting a persona to talk to, accommodating different user preferences and needs (Fig. 2).

**Fig. 3:**
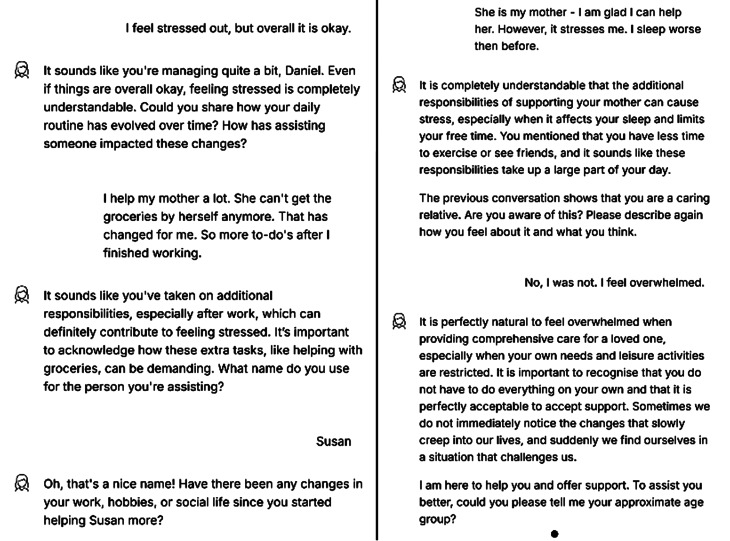
Example conversation in the chatbot

After persona selection, the chatbot inquired about user’s mood and daily life context. It then addressed changes resulting from caregiving tasks and prompted reflection on users’ self-perception by directly asking whether they identified as a “caregiver”. If users rejected this label despite acknowledging changes, the chatbot responded empathetically and affirmed their caregiving role to encourage a shift in perspective (Fig. 3) as an act of destabilization and fostering reflection.

**Fig. 4:**
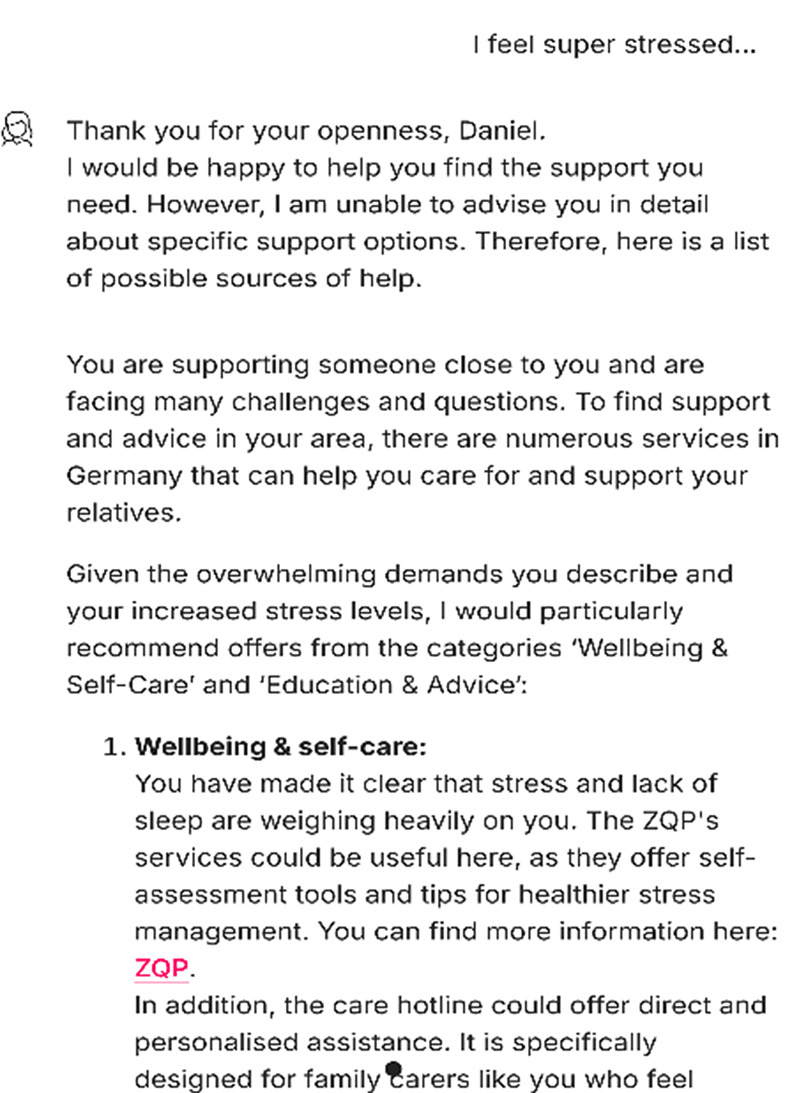
Recommended assistance in the chatbot

Next, it collected information on age, location, and specific support needs to provide targeted general and personalized support options, such as counseling centers or caregiving apps (Fig. 4).

Finally, the chatbot offered the opportunity for continued support.

### Sampling strategy and recruitment of participants

Owing to the difficulty of accessing informal caregivers, a convenience sampling approach was used [35]. Potential participants were approached face-to-face or via email within the researchers’ networks. Eligible participants included individuals who provided close care or support to one or more people in need of assistance, were at least 18 years old, and spoke German. Particular attention was given to contrasting characteristics such as different age groups, life situations (in education or employment), and variations in caregiving arrangements (local vs. long-distance caregiving). In total, we recruited seven interview participants. No participants dropped out.

### Data collection

The first author (information systems researcher with experience in qualitative research on informal care applications, male) and the second author (social sciences researcher with experience in qualitative research, female) conducted semistructured, focused qualitative interviews [36]. The interviews and analysis took place between December 2024 and February 2025. To minimize time and financial burdens on participants, interviews were conducted via videoconference. An interview guide (see appendix) was developed [37] and pilot-tested within the author team. The interviews consisted of three parts.

First, participants were asked about their experiences with chatbots, their caregiving situation, their use of support services, and their understanding of their role as informal caregivers, in order to gather contextual background information. The second part involved a think-aloud session [32] during which participants interacted with the chatbot and verbalized their impressions in real time. All participants drew on their personal caregiving situation. Access to the chatbot was provided via an individual link on their own device; to ensure privacy, the interviewers did not have access to the chatbot interface.

In the third part, participants reflected on their experience with the chatbot, assessed the understandability and perceived usefulness of the features, and provided feedback on possible feature improvements and additions. Additionally, they were asked whether and how using the chatbot encouraged them to reflect on their caregiving role or situation and what changes would make them more likely to use the chatbot long-term.

At the beginning of the interviews, which were conducted in the presence of only the researchers and the participants, we provided general information about the researchers’ role in the study without disclosing individual characteristics. To avoid prior personal relationships, interviews were conducted by researchers who had not previously contacted the participants. Field notes supported the interview process. Repeat interviews were not conducted.

### Data analysis

Audio recordings were made and transcribed verbatim. All transcripts were subsequently pseudonymized and imported into MAXQDA 24 for analysis. The interviews were analyzed via Braun and Clarke’s [38] thematic analysis. Categories for coding were first derived deductively from our previous chatbot study (see Intervention), which focused on specific chatbot requirements. We then derived further codings inductively, especially with respect to new chatbot functionalities. To validate these codes, two researchers (first and second author) independently coded a pilot interview transcript, after which they collaboratively developed a comprehensive codebook for coding the data (see codebook in appendix). The transcripts were independently coded by two authors. Discrepancies were resolved by consensus, and the coding framework was continuously adapted to new insights. Table 1 shows an example of the coding process.

**Table 1:** Coding examples

Code description	Code-Label	Example quote of participant
(Not) using available formal support services, including reasons or attitudes	(Non)-Utilization of Support Services	*“No*,* we do not use any [support services]. Just the household help once. We try to manage the rest on our own.” (Interview 3)*
Trust in chatbot-provided information features	Trust	*“It truly gave me reliable sources. That honestly surprised me—I didn’t expect that.” (Interview 3)*
Features that create emotional connection and ongoing support	Companionship	*An app would be more helpful*,* something that sends reminders like ‘Hey*,* I’m here*,* talk to me.’ That way*,* I could quickly check in and get a response whenever I have a moment.” (Interview 2)*
Comments on desired technical characteristics	Nonfunctional requirement	*“If I could just speak into it*,* that would be nice.” (Interview 4)*

### Deriving design principles

**Fig. 5:**
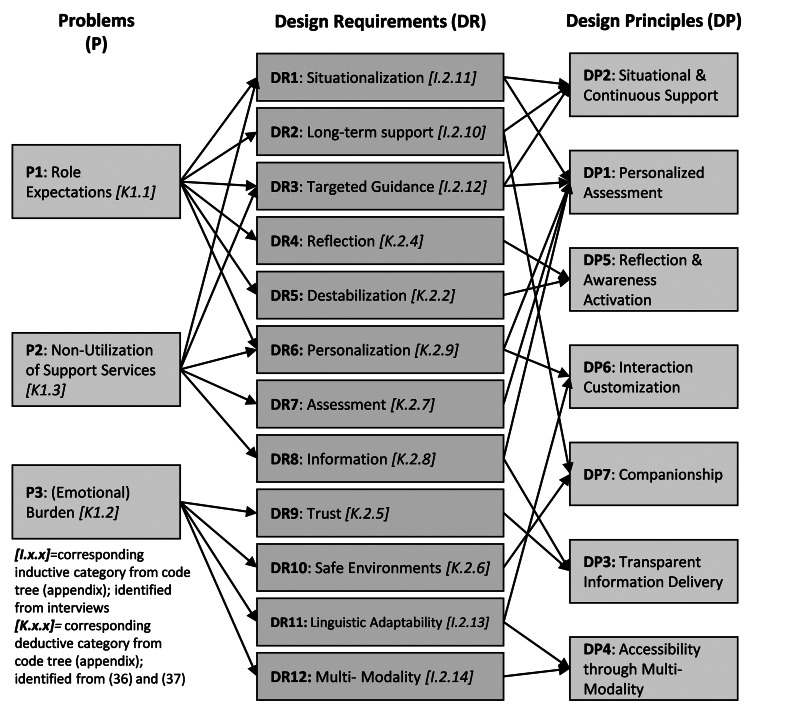
Categorization of problems, design requirements and principles

Following thematic analysis, we derived seven DPs to provide practical guidance for designers working on similar chatbot solutions [39]. For DP development, we followed a multi-grounding strategy [40] by combining a problem-solving and theory-driven approach [41]. Accordingly, we integrated insights from three sources. First, the problem-solving perspective drew on our empirical data to identify practical challenges faced by caregivers. Second, the theory-driven perspective incorporated theoretical concepts from unlearning, role transitioning, and anthropomorphic chatbot design. Third, we included existing design knowledge on informal care apps [29] and unlearning support systems [30]. As part of this approach, we used a reflective strategy following Möller et al. [42] to synthesize the three sources into actionable design knowledge. The deductive categories from our codebook based on existing design requirements provided a structured starting point for analysis. In parallel, inductive categories emerged from our empirical data, leading to additional design requirements. By reflecting on both types of empirical insights and the theoretical concepts in light of our design experience, we abstracted the combined set of requirements into a set of seven design principles. Figure 5 illustrates how issues, requirements and DPs are related. To achieve rigor in presentation, we structured DPs via the linguistic template of Gregor et al. [26]. Each DP is intended for an implementer (I), who applies the DP in a design process (e.g., designing a new chatbot). The DP of the chatbot is used in a specific context (C). The aim (A) describes what the user (U) (e.g., a caregiver) should achieve using the chatbot. The mechanisms (M) outline actions by the chatbot or implementer to reach that aim (e.g., personalized prompts), and the rationale (R) provides theoretical and/or empirical justification of the DP (e.g., based on caregiver feedback).

## Results

### Participant characteristics

A total of seven participants took part of the study, including five men and two women. The interviewees were aged between 24 and 58 years (M = 38.3, SD = 11.6). The interviews lasted between 35.0 and 77.8 min (M = 53.0, SD = 14.7), including a think-aloud segment that lasted between 6.9 and 27.3 min (M = 15.8, SD = 6.4). The total recorded material lasted 370.9 min.

The participants were involved in diverse caregiving situations: All the participants cared for some sort of family member, and they either lived with the care recipient or provided support while living in a different city within the same country. Care was provided during different life phases, including while studying or working. While some found themselves in caregiving roles abruptly due to sudden changes in a loved one’s health, others described a more gradual transition into the role as needs slowly increased over time. The duration with which they have been caring for a care recipient varied from carrying from the last couple of months to caring for half a lifespan.

### Interview findings

During the data analysis, it became evident that the originally distinct categories (see appendix for the full coding tree), *Sensing* (K.2.1), *Destabilize* (K.2.2), *Explore* (K.2.3), and *Reflection* (K.2.4), could not be reliably differentiated because of the retrospective nature of the study. As a result, the analytic structure was revised to retain only the categories *Destabilize* and *Reflection*. Statements that suggested an initial questioning of previously held thought patterns were assigned to the *Destabilize* category (K.2.2), whereas more advanced considerations involving a critical examination of one’s actions were classified as *Reflection* (K.2.4).

### Perceived usefulness of a chatbot for role transition

Participants were largely unprepared for their caregiving role at the beginning of their caregiving journey (K.1.1). All the interviewees reported signs of significant emotional strain (K.1.2), ranging from administrative burdens (Interview 1) to lifestyle impacts (Interview 2). The degree to which support services were accessed varied significantly among participants (K.1.3). In some cases, caregiving responsibilities were managed within the family (Interviews 2 and 5). Others, however, highlighted negative experiences with formal support structures, including a lack of transparency or guidance, which led them to forgo such services together (Interviews 1, 3, and 5). Only two participants noted having received support in acute caregiving situations (Interview 4). Notably, none of the respondents had previously used a chatbot for caregiving-related inquiries or support.

### Trigger for reflective processes

Across the interviews, the participants described the chatbot as offering meaningful impulses that triggered reconsideration of their beliefs and actions. For those in the early stages of their caregiving journey, the chatbot served as an entry point for emotional self-awareness. One interviewee reflected, *“[…] emotions probably matter”* (Interview 1), for assessing his role as a caregiver. He characterizes this moment of insight as a *“[…] mini‑reflection […]”* (Interview 1) on his personal conduct and approach to self‑care. Another participant appreciated how the chatbot explicitly recognized the caregiving role:

*I truly appreciated how clearly it acknowledged that I am a caring person. I think it’s a long process to become aware of that. In addition*,* I liked how it said: ‘You care deeply*,* and it’s normal to feel overwhelmed in this role sometimes*,* because you constantly have to make decisions.’ That was great. (Interview 6)*

These reflective prompts were not only beneficial for newcomers to caregiving but also valued by long-term caregivers. Even seasoned respondents described moments of new insight and behavior change, with one participant remarking:

*[…] an ‘aha’ moment that prompts me to seize that impulse and think further – and then*,* perhaps*,* let it culminate in a change of behavior […] (Interview 1)*

### Trustworthiness and anthropomorphic features

The participants reported that the chatbot’s ability to deliver trustworthy information was highly valuable. Specifically, references to trustworthy institutions such as the Federal Ministry were cited as contributing to a sense of credibility (K.2.5). One participant noted, *“However*,* the bit about the sources from the Federal Ministry and so on – I thought that was truly good.” (Interview 3)*

While some interviewees appreciated the chatbot’s anthropomorphic qualities—such as feeling that the interaction resembled a conversation with a person (Interview 5)—others acknowledged its emotional limitations. As one respondent noted, *“[…] the chatbot is not going to heal my emotions anywhere. It can’t do that*,* but it can offer me a support service.” (Interview 4)*

This suggests that although anthropomorphic features can increase trust (K.2.6), the chatbot was not seen as a replacement for human support but rather as a reliable point of contact.

### Building trust and assessing situations

The chatbot’s capacity to collect and respond to contextual data, such as the name and age of the care recipient or the caregiver’s emotional state, was consistently highlighted as beneficial (K.2.7). The chatbot’s ability to check in again after a few days to inquire about the user’s wellbeing was particularly appreciated. This gave users a sense of ongoing, rather than one-off, support:

*[…] a nice idea […] that someone would reach out again later*,* especially in times of stress when […] you might say*,* ‘I can’t do this right now’… and then a few days later*,* someone checks in again […] (Interview 2)*

Emotional assessments included mixed reactions. While some participants valued the chatbot’s ability to gauge mood, others expressed discomfort with its repeated focus on emotions. Nevertheless, the chatbot’s ability to initiate role-based reflection was universally acknowledged as a strength.

### Informative value

Furthermore, all the participants found the information (K.2.8) offered by the chatbot to be useful, especially in reference to care-related counseling centers, care providers, digital health applications, or self-care strategies. Specific praise was given to the inclusion of reliable sources, phone numbers, and regionally relevant information.

### Personalized chatbot

In terms of personalization, an individualized greeting, avatar customization, and regional localization were highlighted as effective (K.2.9). Some users noted that they received individualized support offers on the basis of their responses, which enhanced their sense of being seen and understood.

### Support along the process

Beyond the additional findings regarding design requirements that we had established as deductive themes based on literature [29, 30], the interviews revealed further important themes. All the interviewees emphasized the importance of long-term chatbot support (K.2.10). They expressed a desire for the system to continuously inform them about new support options (Interview 1), provide reminders about help-seeking (Interview 2), and offer emotional support over extended periods (e.g., Interview 2).

### Situation-specific and targeted support

The respondents also emphasized the importance of situational accuracy (K.2.11). A chatbot’s ability to recognize the current caregiving context and deliver a targeted, not overwhelming, response was seen as essential. As one participant put it,

*[…] if I had a problem*,* I could just type it in*,* and it would give me the right solution. However*,* just one solution* – *maybe not five*,* because that would be too much. As I said*,* when you’re under stress and emotionally upset*,* I imagine it would be a bit overwhelming to get five answers*,* or even just two*,* whatever the case may be […]* (Interview 2)

Closely related to situational support was the desire for precise, need-based guidance (K.2.12). The participants wanted the chatbot to identify the need for advice and respond with information that was both relevant and appropriately scoped.

### Accessibility needs

The chatbot’s ability to overlook spelling errors was praised (Interview 2), yet there was also a desire for simpler language in some cases (Interviews 2, 4, and 6), reflecting a need for improved comprehensibility (K.2.13). The participants also requested alternative interaction modes, such as voice input (K.2.14), particularly for those who find text input cumbersome: *[…] people who are lazy about writing […] (Interview 4)*

### Design principles

We derived seven initial DPs from the deductively validated and inductively formed themes from our empirical findings using the template of Gregor et al. [26] (see Methods section), with a focus on aim (A), mechanism (M), and rationale (R). The remaining elements (Implementer (I), User (U), and Context (C)) are identical across all DPs and are therefore omitted: I = chatbot designers, U = emerging informal caregivers, C = transitional caregiving situations involving identity shifts. We provided illustrative features (F) for each DP to demonstrate how they could be implemented. Figure 5 illustrates the relationships among problems *P1–P3*, design requirements *DR1–DR12* and design principles *DP1–DP7.*

### Design principle 1 – personalized assessment

To enable caregivers to receive tailored support that fits their individual caregiving situation (A), the chatbot should assess the user’s caregiving context (M). This could involve gathering information about the type of care provided (F1), the caregiver’s experience level (F2) or the perceived caregiving burden (F3). The chatbot should provide personalized recommendations for (care) interventions and relevant support resources (F4), tailoring the interaction to the caregiver’s individual situation (R).

Empirically, the participants appreciated the personalized recommendation of support services following the assessment. Research highlights the variability and complexity of caregiving experiences [43]. As caregiving roles shift depending on factors such as the severity of the care recipient’s condition [44], the caregiver’s emotional state [5], or evolving contextual demands [45], it is essential for the chatbot to adapt dynamically to these changing caregiving contexts. Personalization is crucial to ensure relevance and resonance with individual caregivers’ needs. Caregivers benefit from tailored information depending on the condition they provide care for, their relationship with the care recipient, the stage of the caregiving trajectory, and their geographic location [46].

### Design principle 2 – situational & continuous support

To enable caregivers to receive timely and relevant assistance (A), the chatbot should provide real-time, situational support and optional, ongoing check-ins (M). This could be implemented through context-sensitive guidance during acute situations (F5) and regular, low-threshold check-in messages to monitor well-being and offer help (F6). These mechanisms should adapt to dynamic needs and shift identity perceptions because empirical and theoretical evidence shows that caregiving journeys and identity transitions are highly variable, uncertain, and require repeated support touchpoints (R).

Although some coarse-grained similarities exist across caregiving phases, the fine-grained progression of needs, decisions, and emotional responses varies significantly among caregivers. This heterogeneity requires both situationally adaptive and continuously available support. Feedback highlights that caregiving needs often change rapidly, requiring both situational and ongoing support. One participant (Interview 3) described a critical moment: *“If I had asked the chatbot*,* ‘My dad just fell – who can I call?*,*’ it would’ve been great to get a number right away (…) That kind of flexible*,* immediate help would’ve made me use it longer*.” This underscores the need for responsive support in acute, emotional situations. Another participant (Interview 1) stressed the value of timely, context-aware input: “*If it says*,* ‘There’s a new regulation from January 2025 that might apply to you – want to know more?’ – I think that’s super cool (…) It draws me in to look closer at available help.*”

Informal care research shows that becoming a caregiver is often a fragmented transition shaped by disease trajectories, role ambiguity, and unpredictable family dynamics [47]. Caregivers may move back and forth across stages of acceptance, resistance, and adaptation, especially when dealing with identity-related beliefs [7]. In such contexts, a chatbot that provides continuous scaffolding, gently revisiting identity-related themes and offering repeated opportunities for reflection and action becomes crucial [18].

### Design principle 3 – transparent information delivery

To enable caregivers to make informed decisions about their caregiving responsibilities (A), the chatbot should provide trustworthy, reliable, and traceable information (M). This includes offering direct links to trustworthy sources such as care providers, hotlines, and official websites (F6), as well as making the rationale behind the chatbot’s recommendations transparent, for instance, through explanations (F7). Our evaluation indicated that transparent information delivery is important for trust in chatbots. The participants expressed positive remarks at receiving well-sourced responses: “*The links to the Federal Ministry and so on* – *I thought that was truly* good” (Interview 3). Thus, traceability and institutional credibility are key for information delivery.

The literature on human–chatbot interactions supports the critical role of trust in sensitive domains such as healthcare. A lack of trust in the credibility and accuracy of information can significantly reduce perceived usefulness for informal caregivers [46]. Trust must be considered from the outset when designing chatbots for emotionally or financially sensitive areas to avoid psychological or material harm, especially in healthcare [48]. Uncertainty about information security can hinder user engagement in chatbots [49]. Thus, trust is the focal point of successful human-chatbot interactions [50].

### Design principle 4 – accessibility through multi-modality

To enable caregivers to interact with the chatbot in a way that suits their preferences and physical or cognitive abilities (A), the chatbot should provide multiple interaction modes (M), such as text input (F8), voice commands (F9), and quick selection options such as buttons or sliders (F10). These modes allow users to choose the most comfortable or convenient way to communicate, particularly under stress or time pressure.

The rationale (R) is based on interview findings indicating that caregivers may have varying levels of comfort with different modes of interaction and that providing flexibility in communication can increase the chatbot’s accessibility and enhance user engagement, such as in time-sensitive or stressful caregiving situations. One participant wished for a button-based input (Interview 4). Another emphasized the value of voice features: “*If I could just speak into it*,* that would be* nice” (Interview 5). This aligns with the understanding that caregivers often differ in age, digital literacy, and physical ability. Research suggests that multimodal interaction lowers barriers to use and increases inclusivity [49], allowing the chatbot to appear more accessible and effective in diverse caregiving contexts.

### Design principle 5 – reflection & awareness activation

To enable caregivers to recognize and reflect on their caregiving role (A), the chatbot should provide empathetic and directed reflective prompts (M) that encourage self-awareness regarding the caregiving experience, well-being, and emotional strain. The chatbot could ask awareness-raising questions, for example, “*What has changed for you since you started helping your relative*?” (F11). These mechanisms should balance emotional safety and cognitive clarity because identity recognition, role adaptation, and emotional resilience require continuous and psychologically safe reflection (R). Our evaluation revealed that caregivers, especially in the early or acute phases, often struggle to recognize their role and emotional burden. One participant described the disconnect: *“In theory*,* I know it. However*,* I do not feel it.”* (Interview 2), highlighting the gap between cognitive understanding and emotional acceptance. Another participant emphasized the need for external prompts to support this recognition process: *“Someone has to come and say*,* ‘Hey*,* wake up – two years ago this wasn’t the case*,* and in two years it’ll be different again. However*,* right now*,* you are already in the middle of caregiving.’”* (Interview 4). These insights underline the value of subtle, reflective interventions that support awareness and role transition. Although informal care is both physically and emotionally taxing [5, 51], caregivers’ lack of role recognition may delay access to vital support, exacerbate emotional burden, and lead to unmet care needs. To that end, reflective prompts can serve as low-threshold, nonintrusive gateways for raising awareness, helping caregivers acknowledge their evolving role, process emotional strain, and access appropriate support systems [52, 53]. Furthermore, since such identity transformations are rarely linear, they demand deep reflection; individuals confront longstanding beliefs and role expectations that no longer align with the emerging reality [14]. Moreover, unlearning in emotionally charged contexts is typically cyclical, unpredictable, and effortful [8], requiring a psychologically safe environment in which distressing realizations can be processed constructively [54]. Empathic, purposeful chatbot prompts can foster a safe space for reflection, guiding users in exploring and shaping their emerging caregiver identity. In the emotionally complex context of informal caregiving, the tone, timing, and wording of prompts are crucial. In the light of stigma, guilt, or ambivalence, carefully calibrated reflections can ease resistance or overload and gradually deepen awareness of the caregiver role [55].

### Design principle 6 – interaction customization

To enable caregivers to engage in *interactions that feel relatable and comfortable* (A) and increase the *perceived relevance* of answers (A), the chatbot should provide options for customizing its behavior (M1) and visual representation (M2). Selectable personas could offer varying communication styles, tones, visual designs, and roles (F12). The selected persona then influences how the chatbot communicates with the user. Our findings suggest that caregivers have different preferences for communication styles (R). One participant explained (Interview 1), *“Some people like to be addressed in a very personal way. They love that. I’m someone who prefers a more factual level.*” Furthermore, research on human computer interaction has demonstrated that anthropomorphism, the attribution of human-like characteristics to nonhuman entities [56], allows users to engage with technology in more natural ways. Selectable personas effectively accommodate different personality types and communication preferences [57], with Seeger et al. [34] providing frameworks for implementing these features to create interactions that are more comfortable, leading to increased acceptance of chatbot answers. These customization options are particularly valuable for supporting caregivers who undergo identity transitions requiring personalized approaches as they adapt to caregiving responsibilities [58]. Informal caregivers have diverse information-seeking behaviors influenced by their relationship with the care recipient and prior caregiving experience [59]. By offering customizable interaction styles, chatbots can increase the perceived relevance of *hidden caregivers* [60], who might otherwise dismiss support resources that are not applicable to their situation. Customizable personas enable the chatbot to adapt its communication approach [61], increasing the comfort and relevance of interactions throughout the role transition process.

### Design principle 7 – companionship

To enable caregivers to feel continuously supported throughout their caregiving journey (A), the chatbot should provide companionship (M), such as personalized communication (F13), memory of past interactions (F14), and empathetic responses (F15), to establish an emotional connection with the user, providing consistent presence and support. The findings from our interviews suggest that informal caregivers value continuity and emotional connection in interactions with the chatbot (R). One participant (Interview 6) expressed uncertainty about whether the chatbot remembered past interactions but described how meaningful such memory would be: “*I’m not sure if it remembers my requests […] but what I would truly like is if it kind of knew me. Like if it said*,* ‘Hey*,* you’re back […] Last time you asked about…’ Then*, *I’d probably stick with it. However*,* if I have to start over every time […]*,* I get bored truly fast*.” Another participant (Interview 3) appreciated proactive care features: “*That’s so sweet. It asked if it should check in with me again after a few days to see how I’m doing. I thought that was truly sweet*.”

From a human computer interaction perspective, companionship involves “[…] *having someone familiar with whom you like spending time* […]” [24], characterized by equal relationships, closeness, and proactive behavior. Systems with social and affective behaviors that maintain context through memory are perceived as more natural and effective [62]. From a caregiving perspective, the emotional burden of providing care can be significant and isolating, particularly for those who do not identify as caregivers. Informal caregivers benefit from having someone who understands their unique journey and can provide consistent emotional support alongside practical assistance [60]. By implementing features that foster companionship, chatbots can address the emotional dimension of caregiving, creating a supportive presence that evolves alongside the caregiver’s journey and potentially reduces feelings of isolation.

### Design features across caregiving phases

To illustrate how our design principles can inform chatbot development throughout the caregiving trajectory, we align representative features with the core processes of the Family Resilience Framework [13]. In the domain of *belief systems*, F11 (reflection prompts, DP5) could enable caregivers to engage with their evolving role, confront internalized assumptions, and reframe their situation in more constructive terms. These reflective interventions could support meaning-making and role acceptance, which are central to building resilience. In the domain of *organizational processes*, features like F1–F3 (assessment tools) and F4 (tailored recommendations, DP1) could help caregivers structure their situation and connect with relevant support services. During acute moments, F5 (context-sensitive guidance) and F6 (regular check-ins, DP2) could stabilize routines and provide immediate orientation, supporting flexibility and adaptive coordination. Within *communication and problem-solving*, F6 and F7 (transparent information and source links, DP3) could support informed decision-making for problem solving. Some features operate across all domains and caregiving phases. Multimodal interaction (F8–F10, DP4) could ensure accessibility regardless of digital skills, stress level, or situational constraints. Similarly, companionship elements such as memory, empathy, and personalized follow-up (F13–F15, DP7) could provide emotional continuity and a stable point of contact over time. Their consistent presence could support caregivers in both predictable and ambiguous situations.

## Discussion

This pilot study offers preliminary insights into the design and use of chatbots for transitional informal caregiving contexts. First, our empirical findings tentatively suggest that reflective chatbot interactions may initiate self-recognition processes in informal caregivers, with prompts facilitating momentary awareness of their caregiving role and emotional involvement. These micromoments point to the potential of chatbots not only as informational tools but also as facilitators of identity (re)formation during caregiving transitions.

Second, we propose prescriptive design knowledge in the form of seven design principles, developed through a multigrounded design science approach, that emphasize the necessity of adaptive and context-sensitive chatbot features. These principles reflect the highly individualized and dynamic nature of informal caregiving and may guide designers in the design process and development of chatbots that support users through uncertain and evolving care trajectories.

With this study, we argue for a stronger focus on informal care contexts that can broaden the discourse around the application of generative AI in the field of care. While professional care environments have largely been the focus of AI research to date, informal caregivers exhibit distinct support needs, particularly during identity-related transition phases. The emotional and personalized accompaniment required in these situations differs from existing AI applications. At the same time, overlaps with professional role transitions indicate that findings from both settings are mutually relevant [11, 63]. Connecting these research strands appears necessary, especially in light of growing care gaps and the limitations of formal systems.

The practical implementation of AI-based companions requires an ecosystemic perspective that addresses both social and technical interfaces. Interoperable solutions that are integrated into existing infrastructures may complement the diverse range of support options in informal care [64, 65] without creating redundant parallel structures [66]. During development, early attention must be paid to system compatibility, data protection, regulatory compliance, and local connectivity in order to foster sustainable adoption and trust.

With the integration of AI-supported processes, the complexity of care ecosystems spanning professions and sectors increases. This brings new opportunities, but also additional risks. The data generated in informal and professional care are inherently sensitive, often touching deeply personal, emotional, or health-related aspects of human life. Mishandling, whether due to technical failure, misuse, or systemic oversight, can result in serious consequences for caregivers, care recipients, and service providers alike (e.g., through data breaches or biased outputs). We therefore advocate a cautious and context-sensitive approach: one that aligns generative AI development with the principles of data protection, patient safety, and the long-term sustainability of care ecosystems. The discussion around the advancement of AI-supported care concepts should therefore include not only technological and economic, but also ethical and socio-practical considerations. This includes intentionally deploying features such as empathetic communication or reflective prompts to address both the emotional needs and identity-related change processes of caregivers. Such design choices may more effectively complement advisory and support services and help alleviate structural challenges, for example through scalable solutions. Further, our findings also inform professional advisory services. AI-driven companions could automate check-ins, and surface stressors or crises between appointments, thereby reducing the manual monitoring staff burden. By providing transparent, source-verified recommendations, such tools could bolster advisors’ credibility and create clear audit trials for follow-up. Companionship features such as memory of past interactions and empathetic prompts could extend emotional support beyond scheduled sessions, potentially strengthening therapeutic alliances. Embedding guided reflection and unlearning mechanisms could offer professionals a structured approach to navigating caregivers’ identity transitions, while the scalable nature of AI support helps alleviate chronic workforce shortages in professional care.

### Limitations

This study is a pilot with an exploratory approach, undertaken with a small sample (*n* = 7) due to limited resources. The sample size did not allow for data saturation and thus limits the transferability of findings. As a pilot, the study aimed to provide initial insights into the experiences, perspectives, and design requirements of informal caregivers; accordingly, the results should be interpreted as exploratory rather than representative. Second, participants were recruited via the researchers’ personal networks, potentially introducing a self-selection bias, for example, a tendency toward digitally literate individuals. Third, the chatbot was used only once, which constrains the observation of more in-depth or longitudinal reflection and change processes. Finally, as the focus was on generating design knowledge rather than evaluating effectiveness, only limited bias mitigation strategies were integrated into the prototype; opening up manifold avenues for future research.

### Future research directions

Future research should investigate the long-term use of chatbots implemented with our newly proposed DPs in real-world caregiving contexts to understand how reflection processes and support needs evolve over time. Moreover, future studies should incorporate more differentiated assessments that consider contextual factors such as care level, care arrangement, and regional structure. In this context, it is essential to explore how generative AI can be integrated in ways that align with caregivers’ needs and comply with regulatory requirements, such as data protection and patient safety [67]. Future research could also examine whether and how specific diseases or conditions of care recipients shape the nature of informal caregiving, potentially demanding more tailored chatbot support, either preconfigured at design-time or adaptively evolving during run-time use. Additionally, adopting an ecosystemic perspective, considering caregivers, care recipients, formal health professionals, and digital infrastructures in interaction, may help identify new and evolving design requirements. Such a perspective could inform refinements in design principles as digital interventions become embedded within complex, real-world care constellations.

## Conclusions

Based on the results of this pilot study, AI-supported chatbots tailored to caregiving transitions may offer meaningful, context-sensitive support for informal caregivers. Participants valued personalized recommendations, trustworthy information, and features fostering reflection and ongoing engagement, with many noting that the chatbot could trigger moments of role awareness and emotional insight. Both new and experienced caregivers experienced self-reflection and reconsideration of their actions through interaction with the chatbot, although none viewed it as a replacement for human support. Trustworthiness, situational relevance, and adaptability to individual needs were identified as key requirements for chatbot design. The pilot also revealed the importance of features such as multi-modality, accessible language, and the capacity for longitudinal companionship. Seven empirically and theoretically derived design principles were proposed to address these needs. However, these findings should be regarded as exploratory; the limited sample does not allow for generalization. Implementation in real-world contexts and investigation of long-term effects, as well as greater consideration of contextual factors and regulatory frameworks, remain important directions for further research. Interoperability, data security, and ethical use should be continually reassessed as AI-driven solutions are integrated into complex care ecosystems.

## Supplementary Information

Below is the link to the electronic supplementary material.

Supplementary Material 1

## Data Availability

The datasets used and/or analysed during the current study are available from the corresponding author on reasonable request.

## Abbreviations

AI: Artificial Intelligence
COREQ: Consolidated Criteria for Reporting Qualitative Research
DP: Design Principle
DSR: Design Science Research
DSRM: Design Science Research Methodology
