# Supplementary material for: Designing an AI companion to support informal caregivers in role transition: insights from a design science approach
Source: BMC Nurs. 2025 Sep 10;24:1165. doi: 10.1186/s12912-025-03868-2 (PMC12424210; doi:10.1186/s12912-025-03868-2)
Supplement: Supplementary file 1 — Supplementary Material 1 [file 12912_2025_3868_MOESM1_ESM.docx]

Multimedia Appendix

***Table 1:*** *Interview Guide as used for the interviews*

| **Interview Guide – Informal caregivers & Chatbot** | | | | |  |
| --- | --- | --- | --- | --- | --- |
|  |  |  |  |  |  |
|  |  |  |  |  |  |
| Type of Data Collection: | | Focused Interview |  |  |  |
| Data Collection Period: | | 11/2024–02/2025 |  |  |  |
|  |  |  |  |  |  |
| **No.** | **Main Question / Prompt** | **Check Aspects** | **Steering Questions** | |  |
| **1.** | **Perception of the role as a caregiving relative – BEFORE USING THE CHATBOT** | | | |  |
| **1.1** | What has been your experience with chatbots to date? |  |  |  |  |
| **1.2** | You are currently supporting someone close to you. Please describe what this support looks like. | Tasks; Self-assessment; Age? Experience with informal care? Health promotion; Risk minimization; Health; Stress; Role changes | **1.2.1** | When did this support situation begin for you? Can you describe it? |  |
|  |  |  | **1.2.2** | What measures did you take at the beginning of this new situation? |  |
|  |  |  | **1.2.3** | How has your own well-being evolved over the course of providing support? |  |
|  |  |  | **1.2.4** | Which moments have you experienced as stressful? |  |
|  |  |  | **1.2.5** | How has your daily life and that of your family changed because of caregiving tasks? |  |
| **1.3** | Please describe the development of the support situation from its start to today. |  | **1.3.1** | Describe the moment you decided to seek external support. |  |
|  |  |  | **1.3.2** | Which services were helpful? Which were not? |  |
| **1.4** | Which support services do you use to assist the person close to you? | Acceptance of services; Triggers | **1.4.1** | What characterizes a supportive person in general for you, regardless of your situation? What tasks and duties do they perform? |  |
|  |  |  | **1.4.2** | Describe the moment you first consciously perceived yourself as a supportive person. |  |
|  |  |  | **1.4.3** | Do you feel adequately prepared for this role? If not, what did you lack? |  |
|  |  |  | **1.4.4** | How do you describe yourself when you tell others about your situation? |  |
| **1.5** | You are in a support role: How do you describe yourself when you talk to others about this support situation? | Role attribution; Identification; Support services; Feeling overwhelmed? |  |  |  |
|  |  |  |  |  |  |
| **2.** | **Use and feedback on the chatbot (think-aloud session) – AFTER USING THE CHATBOT** | | | |  |
| **2.1** | Tell us about the expectations you had when approaching the chatbot. | Expectations | **2.1.1** | What content did you imagine? |  |
|  |  |  | **2.1.2** | Did you assume the chatbot could change something for you? |  |
| **2.2** | How did you perceive navigation within the chatbot? |  | **2.2.1** | Was the chatbot easy to understand, or were there moments of confusion? |  |
| **2.3** | Which chatbot functions did you find useful? Which not? |  |  |  |  |
| **2.4** | If you could wish for anything: How should the chatbot be changed to better suit you and your situation? |  | **2.4.1** | Would you wish for different content? |  |
|  |  |  | **2.4.2** | What functions would you add, adjust, or remove? |  |
|  |  |  |  |  |  |
| **3.** | **Reflection on personal situation and role** | | | |  |
| **3.1** | To what extent did the chatbot prompt you to reconsider or re-evaluate your situation? | Unlearning process: Destabilization; Experimentation; Letting go of patterns; New behaviors; Beliefs | **3.1.1** | To what extent did the chatbot prompt you to reconsider your situation and your role as a caregiving relative? |  |
|  |  |  | **3.1.2** | To what extent did the chatbot prompt you to question aspects of your daily life or consider doing things differently? |  |
|  |  |  | **3.1.3** | To what extent did the chatbot encourage you to try new approaches? |  |
|  |  |  | **3.1.4** | Tell me how the chatbot influenced your beliefs. |  |
|  |  |  |  |  |  |
| **4.** | **Long-term use and companionship features** | | | |  |
| **4.1** | Describe what the chatbot would need for you to use it long-term. |  | **4.1.1** | What kind of support would you wish for from such a chatbot in the long term? |  |
|  |  |  | **4.1.2** | In your opinion, what does the chatbot need to accompany you and help you access support services? |  |
|  |  |  |  |  |  |
| **5.** | **Conclusion** | | | |  |
| **5.1** | Is there anything else important to you that we haven’t discussed? |  |  |  |  |
|  |  |  |  |  |  |
|  |  |  |  |  |  |

***Table 2:*** *Codebook as used for the thematic analysis*

| **Codebook – Informal caregivers & Chatbot** | | |  |
| --- | --- | --- | --- |
|  |  |  |  |
|  |  |  |  |
| Type of Data Collection: | | Focused Interview |  |
| Data Collection Period: | | 11/2024–01/2025 |  |
|  |  |  |  |
| **Code** | **Category** | **Definition** |  |
| K.1 | Challenges of Caregivers |  |  |
| K.1.1 | Wrong Role Expectations | All statements about false or unrealistic expectations caregivers hold about their role. |  |
| K.1.2 | (Emotional) Burden | All statements about any sort of burden arising from the caregiving situation (e.g., fear, grief, feelings of guilt) |  |
| K.1.3 | Non-Utilization of Support Services | All statements on conscious or unconscious non-use of (external) support services that aid in daily caring work |  |
| K.2 | Design Requirements |  |  |
| K.2.1 | Sensing | All statements on sensations of burdening behaviours |  |
| K.2.2 | Destabilize | All statements on conscious questioning of existing patterns of thought and behavior to initiate |  |
| K.2.3 | Explore | All statements on functions that allow users to explore alternative ways of thinking, roles or alternative ways of action |  |
| K.2.4 | Reflection | All statements on support for self-reflection to critically examine one's role, expectations, and actions critically |  |
| K.2.5 | Trust | All statements on building a trusting relationship between the chatbot and users |  |
| K.2.6 | Safe Environments | All statements on creating a safe digital space without fear of judgment or consequences |  |
| K.2.7 | Assessment | All statements on functions to capture current care situation, mood, or other context-related information |  |
| K.2.8 | Information | All statements on the provision of evidence-based information |  |
| K.2.9 | Personalization | All statements on the adaption of content to individual needs, preferences, and life situations |  |
| K.3 | Perceived Usefulness |  |  |
| K.3.1 | Wrong Role Expectations | All statements on the usefulness of chatbot to recognize and discard unrealistic role expectations |  |
| K.3.2 | (Emotional) Burden | All statements on usefulness of chatbot to reduce caregiver burden |  |
| K.3.3 | Non-Utilization of Support Services | All statements on usefulness of chatbot to encourage use of support services |  |

´***Table 3:*** *Code-Tree as derived from the thematic analysis (including inductively developed categories)*

| **Code-Tree – Informal caregivers & Chatbot** | | |  |
| --- | --- | --- | --- |
|  |  |  |  |
|  |  |  |  |
| **Code** | **Category** | **Definition** |  |
| K.1 | Challenges of Caregivers |  |  |
| K.1.1 | Wrong Role Expectations | All statements about false or unrealistic expectations caregivers hold about their role. |  |
| K.1.2 | (Emotional) Burden | All statements about any sort of burden arising from the caregiving situation (e.g., fear, grief, feelings of guilt). |  |
| K.1.3 | Non-Utilization of Support Services | All statements on conscious or unconscious non-use of (external) support services that aid in daily caring work. |  |
| K.2 | Design Requirements |  |  |
| ~~K.2.1~~ | ~~Sensing~~ | ~~All statements on sensations of burdening behaviours~~ |  |
| K.2.2 | Destabilize | All statements on conscious questioning of existing patterns of thought and behavior to initiate. |  |
| ~~K.2.3~~ | ~~Explore~~ | ~~All statements on functions that allow users to explore alternative ways of thinking, roles or alternative ways of~~ |  |
| K.2.4 | Reflection | All statements on support for self-reflection to critically examine one's role, expectations, and actions critically. |  |
| K.2.5 | Trust | All statements on building a trusting relationship between the chatbot and users. |  |
| K.2.6 | Safe Environments | All statements on creating a safe digital space without fear of judgment or consequences. |  |
| K.2.7 | Assessment | All statements on functions to capture current care situation, mood, or other context-related information. |  |
| K.2.8 | Information | All statements on the provision of evidence-based information. |  |
| K.2.9 | Personalization | All statements on the adaption of content to individual needs, preferences, and life situations. |  |
| I.2.10 | Long-term support | All statements on chatbot support beyond a one-time use scenario. |  |
| I.2.11 | Situationalization | All statements on the fitting of the chatbot's responses to the individual care situation the caregivers are in. |  |
| I.2.12 | Targeted Guidance | All statements on the fitting of the chatbot's responses to the individual support motifs. |  |
| I.2.13 | Linguistic Adaptability | All statements on linguistic aspects of the chatbot's responses. |  |
| I.2.14 | Multi Modality | All statements on the availability of taking assistance by the chatbot through different modes. |  |
| K.3 | Perceived Usefulness |  |  |
| K.3.1 | Wrong Role Expectations | All statements on the usefulness of chatbot to recognize and discard unrealistic role expectations. |  |
| K.3.2 | (Emotional) Burden | All statements on usefulness of chatbot to reduce caregiver burden. |  |
| K.3.3 | Non-Utilization of Support Services | All statements on usefulness of chatbot to encourage use of support services. |  |

**Table 4**: Exemplary proposed design changes of chatbot prototype after evaluation

| **Category** | **Change of chatbot design** | **Interview** |
| --- | --- | --- |
| K.2.7. Assessment | Differentiation between care types and experience levels | 4, 6 |
|  | Supportive language when asking emotional questions to avoid distress | 3 |
|  | More input options beyond free text (e.g., sliders, category selections) | 4, 6 |
| K.2.4. Reflection | Emphasizing the importance of self-care for caregivers | 6 |
|  | Clear explanations of what it means to be an informal caregiver in accessible terms | 6 |
| K.2.8. Information | Provide more sources to tailored information (transparency) | 2 |
|  | More regionalized content, references to care levels and resources, and short summaries with deeper dives | 3, 4 |
|  | Additional informational resources on topics like mental health support and caregiving tasks (e.g., how to assist with bathing) | 3, 4, 5 |
|  | Make advantages of the informational resources visible | 2 |
| K.2.9. Personalization | Region-specific phone numbers and stronger adaptation to care experience of caregivers | 2, 4 |
|  | More explicit integration of previously shared information on care situation | 6 |
| I.2.11. Situalization | Situational support for context-specific help | 2,3,4,5 |
| I.2.14 Multi Modality | Multimodal support, e.g., voice input | 4 |
| I.2.10 Long-Term Support | Schedule reminders for follow-up conversations | 1, 4 |
| I.2.10 Long-Term Support  K.2.6 Safe Environments | Continuity in recommendations | 1 |
|  | Memory of past interactions | 6 |
|  | Ability to share chatbot content with others | 2 |

**Table 5**: Consolidated criteria for reporting qualitative studies (COREQ): 32-item checklist

| **No. Item** | **Guide questions/description** | **Reported on Page #** |
| --- | --- | --- |
| **Domain 1: Research team and reﬂexivity** |  |  |
| *Personal Characteristics* |  |  |
| 1. Interviewer/facilitator | Which author/s conducted the interview or focus group? | 8 |
| 2. Credentials | What were the researcher’s credentials? E.g. PhD, MD | 1 |
| 3. Occupation | What was their occupation at the time of the study? | 8 |
| 4. Gender | Was the researcher male or female? | 8 |
| 5. Experience and training | What experience or training did the researcher have? | 8 |
| *Relationship with participants* |  |  |
| 6. Relationship established | Was a relationship established prior to study commencement? | 9 |
| 7. Participant knowledge of the interviewer | What did the participants know about the researcher? e.g. personal goals, reasons for doing the research | 9 |
| 8. Interviewer characteristics | What characteristics were reported about the inter viewer/facilitator? e.g. Bias, assumptions, reasons and interests in the research topic | 9 |

| **Domain 2: study design** |  |  |
| --- | --- | --- |
| *Theoretical framework* |  |  |
| 9. Methodological orientation and Theory | What methodological orientation was stated to underpin the study? e.g. grounded theory, discourse analysis, ethnography, phenomenology, content analysis | e.g., 4-5 |
| *Participant selection* |  |  |
| 10. Sampling | How were participants selected? e.g. purposive, convenience, consecutive, snowball | 8 |
| 11. Method of approach | How were participants approached? e.g. face-to-face, telephone, mail, email | 8 |
| 12. Sample size | How many participants were in the study? | 8 |
| 13. Non-participation | How many people refused to participate or dropped out? Reasons? | 8 |
| *Setting* |  |  |
| 14. Setting of data collection | Where was the data collected? e.g. home, clinic, workplace | 9 |
| 15. Presence of non-participants | Was anyone else present besides the participants and researchers? | 8 |
| 16. Description of sample | What are the important characteristics of the sample? e.g. demographic data, date | 12 |
| *Data collection* |  |  |
| 17. Interview guide | Were questions, prompts, guides provided by the authors? Was it pilot tested? | 9 |
| 18. Repeat interviews | Were repeat inter views carried out? If yes, how many? | 9 |
| 19. Audio/visual recording | Did the research use audio or visual recording to collect the data? | 9 |
| 20. Field notes | Were ﬁeld notes made during and/or after the interview or focus group? | 6 |
| 21. Duration | What was the duration of the interviews or focus group? | 12 |
| 22. Data saturation | Was data saturation discussed? | 27 |
| 23. Transcripts returned | Were transcripts returned to participants for comment and/or correction? | N/A |
| **Domain 3: analysis and ﬁndings** |  |  |
| *Data analysis* |  |  |
| 24. Number of data coders | How many data coders coded the data? | 10 |
| 25. Description of the coding tree | Did authors provide a description of the coding tree? | Appendix |
| 26. Derivation of themes | Were themes identiﬁed in advance or derived from the data? | 9,10 |
| 27. Software | What software, if applicable, was used to manage the data? | 9 |
| 28. Participant checking | Did participants provide feedback on the ﬁndings? | **N/A** |
| *Reporting* |  |  |
| 29. Quotations presented | Were participant quotations presented to illustrate the themes/ﬁndings? Was each quotation identiﬁed? e.g. participant number | e.g., 10, 13, 14, 15 |
| 30. Data and ﬁndings consistent | Was there consistency between the data presented and the ﬁndings? | **N/A** |
| 31. Clarity of major themes | Were major themes clearly presented in the ﬁndings? | 12-16 |
| 32. Clarity of minor themes | Is there a description of diverse cases or discussion of minor themes? | 12-16 |
